# Clinical competency: a gap in language access training for underserved populations

**DOI:** 10.1080/10872981.2026.2653416

**Published:** 2026-04-01

**Authors:** Ethan Bell

**Affiliations:** a University of Central Florida College of Medicine, Orlando, FL, USA

To the Editors,

The curriculum mapping study by Cottrell and colleagues offers a useful contribution to how medical schools quantify instruction in disability and language access. Their finding that a rural LCME-accredited programme dedicates approximately 177 hours to ESL-related competencies across four years reflects meaningful institutional commitment, and their emphasis on early foundational instruction reinforced through clinical application aligns with evidence for durable skill development [1]. The authors also acknowledge an important boundary of the method: curriculum mapping provides quantitative information on hours and topics, but does not assess the quality of instruction or actual competency development [1].

Functional competency can be described as the observable performance in interpreter-mediated clinical communication [2]. In practical terms, that includes recognising when a qualified interpreter is needed, briefly pre-briefing the interpreter, speaking directly to the patient in the first person, pacing information in short segments, checking understanding with teach-back, documenting interpreter use, and responding appropriately when interpreter access is limited. A student may complete 177 hours of ESL-related instruction, knowing professional interpreters should be used and that ad hoc interpretation carries error risk, without ever conducting a clinical interview through an interpreter, navigating an encounter where none is available, or noticing how ‘accurate’ interpretation can still miss hesitation and affect. Exposure and functional competency are related, but distinct outcomes, and curriculum maps document the former more reliably than the latter. A practical complement would be to demonstrate interpreter-mediated communication competency in observed assessments (e.g., OSCE-style encounters): pre-brief the interpreter, use first-person speech, use teach-back, and document when interpreter access is limited [3].

For institutions with limited resources, assessment does not require a new high-stakes exam. A feasible approach is to embed a brief rubric into an existing communication-skills OSCE, simulation, or clerkship direct-observation exercise. Fune and colleagues used a 16-item yes/no checklist and found improved post-workshop performance [3]. In lower-resource settings, an even smaller tool could work: 6 to 8 core behaviours scored with a binary checklist plus one global rating [3]. Future mapping exercises could capture this more accurately by documenting a small set of assessment fields at the learning-event level: assessment method, setting, assessor, whether performance was directly observed, the behaviours assessed, scoring anchors, and any passing threshold. This would let mapping studies show where language-access content is taught, practiced, and observed.

The authors rightly note that their rural Appalachian context shapes the competencies students need and that community-based experiences reinforce classroom instruction [1]. Rural underserved settings present a specific version of the language-access problem: interpreters are often unavailable, family members step in by default, and time pressure makes ad hoc interpretation feel like the only practical option. Training that gives students structured practice navigating this tension develops skills that description alone cannot.

One addition worth considering is integrating bilingual medical students as trained interpreter peers into clinical skills curricula in supervised educational settings (e.g., simulation/skills training), rather than as substitutes for certified interpreters in clinical care. Programmes that implement structured interpreter training for bilingual medical students report improved interpreting-related skills and gains in humanistic competencies, such as empathy toward patients with limited English proficiency [4]. The rural context the authors describe is precisely where such scalable, competency-based training matters most.

## Data Availability

There is no data associated with this research.
